# Clinical Application of Cine-MRI in the Visual Assessment of Mitral Regurgitation Compared to Echocardiography and Cardiac Catheterization

**DOI:** 10.1371/journal.pone.0040491

**Published:** 2012-07-17

**Authors:** John Heitner, Geetha P. Bhumireddy, Anna Lisa Crowley, Jonathan Weinsaft, Salman A. Haq, Igor Klem, Raymond J. Kim, James G. Jollis

**Affiliations:** 1 Department of Medicine, Division of Cardiology, New York Methodist Hospital, Brooklyn, New York, United States of America; 2 Cardiovascular Magnetic Resonance Center, Duke Medical Center, Durham, North Carolina, United States of America; Brigham and Women’s Hospital, Harvard Medical School, United States of America

## Abstract

**Background:**

Detecting and quantifying the severity of mitral regurgitation is essential for risk stratification and clinical decision-making regarding timing of surgery. Our objective was to assess specific visual parameters by cine-magnetic resonance imaging (MRI) in the determination of the severity of mitral regurgitation and to compare it to previously validated imaging modalities: echocardiography and cardiac ventriculography.

**Methods:**

The study population consisted of 68 patients who underwent a cardiac MRI followed by an echocardiogram within a median time of 2.0 days and 49 of these patients who had a cardiac catheterization, median time of 2.0 days. The inter-rater agreement statistic (Kappa) was used to evaluate the agreement.

**Results:**

There was moderate agreement between cine MRI and Doppler echocardiography in assessing mitral regurgitation severity, with a kappa value of 0.47, confidence interval (CI) 0.29–0.65. There was also fair agreement between cine MRI and cardiac catheterization with a kappa value of 0.36, CI of 0.17–0.55.

**Conclusion:**

Cine MRI offers a reasonable alternative to both Doppler echocardiography and, to a lesser extent, cardiac catheterization for visually assessing the severity of mitral regurgitation with specific visual parameters during routine clinical cardiac MRI.

## Introduction

Long-term outcomes of patients with mitral regurgitation have demonstrated increasing rates of heart failure, atrial fibrillation and sudden cardiac death. [1], [2] The mortality risk has been shown to be directly related to the severity of mitral regurgitation. [3] In addition, the severity of mitral regurgitation is a major determinant that leads to left ventricular dilatation and dysfunction. [4] Thus, detecting and quantifying the severity of mitral regurgitation is essential for risk stratification and clinical decision-making regarding timing of surgery. Echocardiography and cardiac ventriculography are well-validated techniques in assessing the severity of mitral regurgitation. [5], [6] Magnetic resonance imaging (MRI) has been shown to be able to assess mitral regurgitation, however, it lacks the validation that exists with echocardiography and cardiac ventriculography.

There are several MRI methods available to assess the severity of mitral regurgitation. Cine-MRI quantifies mitral regurgitation by comparing the difference between the right and left ventricular volumes in the absence of any other significant valvular regurgitant lesions. [7] Cine-MRI enhances the visual assessment of the severity of regurgitant jet as a signal loss, caused mainly from the turbulence of blood leading to the cancellation of the different phase angles of spins subjected to different velocities and acceleration. Flow velocity mapping can assess the severity of mitral regurgitation by quantifying the amount of blood that regurgitates across the mitral valve by using a special pulse sequence that takes advantage of the movement of blood.

Flow velocity mapping and volumetric assessment have been validated in prior studies comparing it to other imaging modalities, however, they require different pulse sequences and/or extra time in post processing. In a high volume cardiac MRI laboratory, this adds extra time to the overall study, thus, validation of the routine sequences used (cine-MRI) on a normal cardiac assessment for left ventricular function would be very beneficial. Higgins et al., validated the assessment of mitral regurgitation via an older gradient echo pulse sequence using the gradient-recalled acquisition by a steady-state (GRASS) technique. [8] However, there have been no studies to date that have validated assessment of the severity of mitral regurgitation using the newer cine pulse sequence SSFP (Steady-state free precession).

Our objective was to assess specific visual parameters by cine-MRI in the determination of the severity of mitral regurgitation and to compare the visual assessment of mitral regurgitation by cine MRI to previously validated imaging modalities: echocardiography and cardiac ventriculography.

We hypothesized that the visual assessment of mitral regurgitation by cine-MRI will be similar to the assessment of mitral regurgitation by echocardiography and left ventriculography in patients that have undergone all three imaging techniques for clinical assessment.

## Materials and Methods

### Study Population

We retrospectively evaluated patients who had undergone cardiac MRI, echocardiography and cardiac catheterization. A total of 68 patients underwent a cardiac MRI and echocardiogram within a median time of 2.0 days for either congestive heart failure or coronary artery disease. Of these 68 patients, 49 also had a cardiac catheterization, median time of 2.0 days between the cardiac catheterization and MRI. The reason for referral for each study varied greatly, ranging from the assessment of coronary artery disease to the assessment of valvular disease. The patients were chosen based upon retrospective assessment of our database for any patient who underwent both echocardiogram and cardiac MRI within 2 weeks.

### Ethics Statement

The Institutional Review Board (IRB) of Duke University Health System approved the study. A written informed consent was obtained from all the study patients.

### Study Design

**Figure 1:**
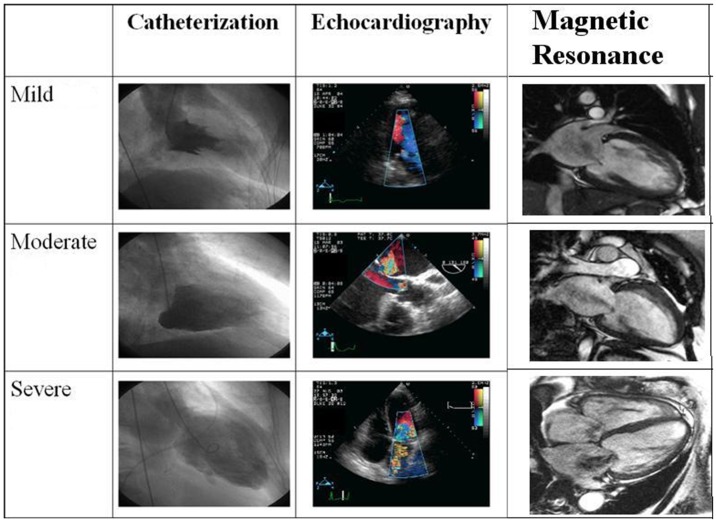
Examples of mild, moderate, and severe mitral regurgitation for each imaging modality.

All studies were reassessed for the severity of mitral regurgitation by consensus of three cardiologists at the same time, who were blinded to the patient data. The visual parameters used in assessing the severity of mitral regurgitation via all three modalities are listed in Table 1. A visual example of each severity for all three imaging modalities is presented in figure 1. Etiologies for mitral regurgitation included myxomatous disease, annular dilatation and ischemic cardiomyopathy.

**Table 1:** Visual assessment parameters for determining mitral regurgitation severity.

Parameters	Severity Mitral Regurgitation		
	Mild	Moderate	Severe
**Cine-Magnetic Resonance Imaging**			
Vena Contracta	<0.2 cm	>0.2 to <0.5 cm	>0.5 cm
Jet Intensity	Iso-intense	Dark grey	Black severe
Jet Area	<20% LA area	>20% to <40% LA area	>40% LA area
Jet Length	<1/3 of LA	>1/3 to <2/3 of LA	>40% LA area
LA Size	<4.0 cm	>4.0 to <5.5 cm	>5.5 cm
LVESD	-	-	>4.5 cm
**Ventriculogram ** [**1**]			
LA Opacification	Opacification < LV Opacification	LA Opacification = LV Opacification	LA Opacification > LV Opacification
**Echocardiography ** [**2**]			
Vena Contracta	<0.3 cm	0.3 to 0.69 cm	>0.7 cm
Pulmonary Vein Flow	Systolic dominance	Systolic blunting	Systolic reversal
Jet Area	<20% LA area	>20% to <40% LA	>40% LA
LA Size	Normal	Normal to <5.5 cm	>5.5 cm
LVESD	-	-	>4.5 cm

1. Grossman W, Dexter L. Profiles in valvular heart disease. In: Grossman W, ed. *Cardiac Catheterization and Angiography*. 2nd ed. Philadelphia, Pa: Lea & Febiger; 1980: 305–324.

2. Zoghbi WA, Enriquez-Sarano M, Foster E, et al. *Recommendations for evaluation of the severity of native valvular regurgitation with two dimensional and Doppler echocardiography*. J Am Soc Echocardiogr. 2003;16: 777–802.

LA: Left Atrial.

LVESD  =  Left Ventricular End-Systolic Diameter.

### Echocardiography

Complete Doppler echocardiography was performed using a Philips Sonos 7500 ultrasound scanning equipment (Andover, MA). The standard views were obtained including parasternal, apical, and sub-costal views with color, continuous and pulse wave Doppler. The mitral regurgitation was assessed using the comprehensive data from the echocardiogram including assessment of the color Doppler jet (size, vena contracta, zone of convergence, and other characteristics (i.e. central vs. eccentric), continuous wave Doppler across the mitral valve and pulse wave Doppler of the pulmonary veins. [9]–[11] In addition, left atrial size were also assessed by parasternal long axis view. [4].

### MRI

Cardiac MRI was performed on a 1.5 Tesla scanner (Siemens Sonata) using a 6-element cardiac phase–array receiver coil. Steady-state free-precession (SSFP) cine images were acquired in multiple short-axis (every 1 cm through the entire left ventricle, with a slice thickness of 6 mm; gap between slices, 4 mm) and 3 perpendicular long axis views across the mitral valve. Typical parameters used were: repetition time, 3.0 ms; echo time, 1.5 ms; flip angle, 90°; temporal resolution, 35 ms/phase; in-plane resolution, 1.7×1.4 mm.

**Figure 2:**
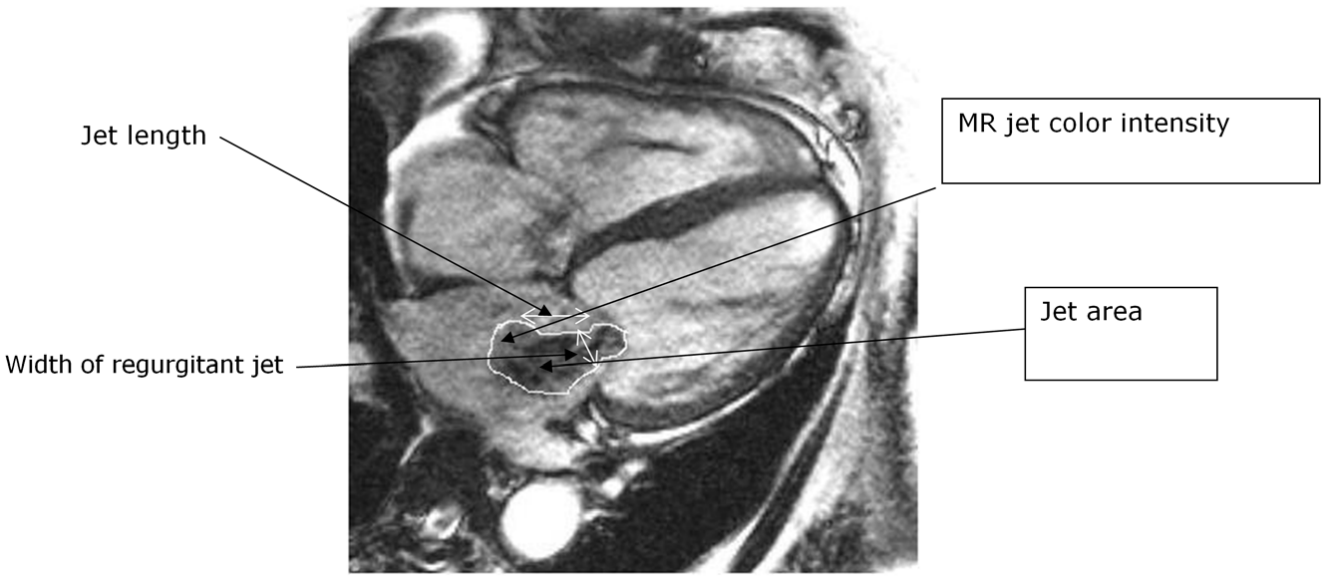
Measurement of visual assessment parameters.

The characteristics used in the assessment of the severity of mitral regurgitation included: vena contracta size, jet intensity, jet area and length, the left atrial size (using a long axis view) and the left ventricular cavitary size (Figure 2). [12] The severity of mitral regurgitation was predominantly based upon the first four of the six criteria listed in table 1. The left atrial size and left ventricular end diastolic diameter was used as additional data in the visual assessment for discriminating the severity of regurgitation. This was an attempt to validate these visual assessment criteria.

### Cardiac Ventriculogram

Cardiac ventriculography was obtained with a 6 F pigtail catheter inserted into the femoral artery and guided into the left ventricle. A power injector was used to fill the left ventricle. Standard ventriculography in the 30 degrees right anterior oblique position was obtained. For patients with an uncertain degree of mitral regurgitation, LAO views were also obtained. The severity of mitral regurgitation was based on assessment of left atrial opacification by Grossman’s criteria (see Table 1), determined by an interventional cardiologist. [13].

### Statistical Analysis

The inter-rater agreement statistic (Kappa) was used to evaluate the agreement between echocardiography and cardiac catheterization, cine-MRI and echocardiography, and cine-MRI and cardiac catheterization. Since kappa does not take into account the degree of disagreement between observers and all disagreement is treated equally as total disagreement, for final analysis we chose to use weighted kappa, and assign different weights to subjects for whom the raters differ by *i* categories, so that different levels of agreement can contribute to the value of Kappa (*K*). The *K* value was interpreted as is shown in Table 2. [14].

**Table 2:** Weighted Kappa ranges.

Value of K	Strength of agreement
<0.20	Poor
0.21–0.40	Fair
0.41–0.60	Moderate
0.61–0.80	Good
0.81–1.00	Very good

## Results

### Baseline Characteristics

The baseline characteristics of the 68 patients are highlighted in Table 3. The mean age was 62±10 years, with 2 out of 3 patients having a history of hypertension. Nearly half of the patients had a history of myocardial infarction and congestive heart failure, with the majority having New York Heart Association (NYHA) class II or III heart failure.

**Table 3:** Baseline characteristics.

Characteristic	Entire Group (n = 68)
Mean Age – yr	62±10
Female	27 (39%)
**Weight (lbs)**	182±24
**Height (ft)**	5.3±1.2
Heart rate (bpm)	78±9
**CAD Risk Factors**	
Diabetes	29 (40%)
Hypertension	45 (66%)
Cigarette Smoker	14 (20%)
**Past Medical History**	
Prior MI	32 (46%)
Prior PCI	16 (23%)
CABG	20 (29%)
ESRD on hemodialysis	5 (7%)
Congestive Heart Failure	33 (49%)
NYHA I	2 (3%)
NYHA II	14 (20%)
NYHA III	15 (21%)
NYHA IV	2 (3%)
CVA	12 (18%)
**LVEDD (cm)**	6.15±1.8
**LVESD (cm)**	4.9±1.4

CAD, coronary artery disease; MI, myocardial infarction; PCI, percutaneous coronary angiography; CABG, coronary artery bypass grafting; ESRD, end stage renal disease; NYHA, new york heart association; CVA, cerebrovascular accident; LVEDD, left ventricular end-diastolic diameter; LVESD, left ventricular end-systolic diameter.

### Cine MRI Compared to Doppler Echocardiography

There was moderate agreement between cine MRI and Doppler echocardiography in assessing mitral regurgitation severity, with a kappa value of 0.47, confidence interval (CI) 0.29–0.65 (Table 4). Of the 29 patients diagnosed with mild or no mitral regurgitation by cine MRI, 28 of them had the same findings by echocardiography. Of the 22 patients diagnosed as having moderate or severe mitral regurgitation by Doppler echocardiography, 21 patients were found to have moderate or severe mitral regurgitation by MRI. Because of small sample size, only data for moderate and severe mitral regurgitation was reported in the tables, which is more relevant for clinical decision-making.

**Table 4:** Comparing MRI and echocardiography.

Table of MRI by Echocardiography							
		Echocardiography					Total
MRI		< Moderate			Moderate or severe		
< **Moderate**		28			1		29
**Moderate or severe**		18			21		39
**Total**		46			22		68
**Kappa Statistics**							
**Statistic**	**Value**		**ASE**	**95% Confidence Limits**			
**Weighted Kappa**	0.469		0.091	0.290		0.648	

### Cine MRI Compared to Cardiac Catheterization

There was fair agreement between cine MRI and cardiac catheterization with a kappa value of 0.36, (CI of 0.17–0.55) (Table 5). Of the 38 patients with mild mitral regurgitation by cardiac catheterization, 21 patients had mild mitral regurgitation as determined by MRI. The remaining 17 patients were diagnosed as having moderate or severe mitral regurgitation by MRI, indicating a higher threshold in assessing the severity of mitral regurgitation by MRI. All 11 patients who had moderate or severe mitral regurgitation by catheterization also had moderate or severe mitral regurgitation by MRI.

**Table 5:** Comparing MRI and cardiac catheterization.

Table of CATH by MRI							
		MRI					Total
Cath		< Moderate			Moderate or severe		
**< Moderate**		21			17		38
**Moderate or severe**		0			11		11
**Total**		21			28		49
**Kappa Statistics**							
**Statistic**	**Value**		**ASE**	**95% Confidence Limits**			
**Weighted Kappa**	0.357		0.097	0.168		0.546	

### Doppler Echocardiography Compared to Cardiac Catheterization

There was good agreement between Doppler echocardiography and cardiac catheterization (kappa value of 0.66, CI 0.42–0.91) (Table 6). Of the 31 patients diagnosed as having mild mitral regurgitation by Doppler echocardiography, only one patient was found to have moderate to severe mitral regurgitation by cardiac catheterization. Furthermore, of the 14 patients with moderate or severe mitral regurgitation diagnosed by Doppler echocardiography, Five patients had mild mitral regurgitation with cardiac catheterization.

**Table 6:** Comparing cardiac catheterization and echocardiography.

Table of CATH by Echocardiography							
	Echocardiography					Total	
Cath	< Moderate			Moderate or severe			
**< Moderate**	30			5		35	
**Moderate or severe**	1			9		10	
**Total**	31			14		45	
**Kappa Statistics**							
**Statistic**		**Value**	**ASE**		**95% Confidence Limits**		
**Weighted Kappa**		0.663	0.124		0.420		0.906

### Stratification Based on Ejection Fraction and Left Atrial Size

To further consider potential mechanisms for discordance, we stratified patients based on left atrial (LA) size and ejection fraction (EF) in determining the severity of mitral regurgitation. When comparing MRI with echocardiography, there was evidence to suggest that the agreement between echocardiography and MRI improved as the left ventricular function declined and no difference was found when stratified for LA size. There was no difference in the severity of mitral regurgitation between MRI and cardiac ventriculography when stratified by EF (either less than or greater than 20%). However, the correlation was worse when stratified by LA size, with greater LA size having a worse correlation for the severity of mitral regurgitation between the two modalities. There was no difference in the assessment of mitral regurgitation between echocardiography and ventriculography when stratified for larger LA size or a lower EF.

## Discussion

The results of our study demonstrate that visual assessment of mitral regurgitation by cine MRI is reasonably comparable to Doppler echocardiography and to a lesser extent cardiac catheterization. There was moderate agreement between MRI and Doppler echocardiography. The correlation between cine MRI and echocardiography was not outstanding, however, cine MRI was able to distinguish severe mitral regurgitation from mild to moderate, which helps in clinical decision making. There was only one patient who was diagnosed with moderate to severe mitral regurgitation by echocardiography who was not determined to have the same severity of mitral regurgitation by MRI. Only one patient with no or mild mitral regurgitation by MRI was found to have moderate mitral regurgitation by echocardiography. There was also a fair agreement between cine MRI and cardiac catheterization in assessing mitral regurgitation severity. Acquisition of multiple 4-chamber long axis views using SSFP in patients with mitral regurgitation seen in any one of the routine views might further increase the agreement.

This study found a good correlation between Doppler echocardiography and cardiac catheterization. These findings are consistent with previously published data validating both of these imaging modalities for assessing the severity of mitral regurgitation. [15]–[18] Mann et al., quantified mitral valve insufficiency in 60 patients and found very good correlation, r = 0.89, between the two modalities in estimating the severity of mitral regurgitation. [17].

MRI identified a higher number of patients with significant (moderate or severe) mitral regurgitation as compared to echocardiography and cardiac catheterization. Of the 39 patients diagnosed as having moderate to severe mitral regurgitation by MRI only 21 of these patients were determined to have similar disease severity by echocardiography. MRI detected all 11 patients who had significant mitral regurgitation by cardiac catheterization. The higher threshold of cine MRI in diagnosing moderate or severe mitral regurgitation as compared to echocardiography and cardiac catheterization may be secondary to the parameters used by MRI. These parameters may need to be adjusted to better correlate with other imaging techniques, as they have not been previously validated. Alternatively, this higher threshold may be more accurate, however outcome data (progression of left ventricular failure, and mortality) would be necessary to confirm this alternative explanation. Furthermore, cine MRI provides detailed information about entire mitral valve apparatus, papillary muscle abnormalities and right ventricular morphology, which has both therapeutic and prognostic implications in patients with mitral regurgitation. [19].

Cine MRI evaluates the severity of mitral regurgitation through the loss of signal created by the turbulence of protons in blood pool created in the left atrium. In patients who have increased left atrial pressures, there may be less of a gradient between the left ventricle and left atrium, which may lead to more severe mitral regurgitation being underestimated due to the lower turbulence created. However, similar pitfall applies to visual estimation of the regurgitant jet by color Doppler echocardiography. When adjusted for left atrial size, there was worse agreement between MRI and echocardiography and cardiac catheterization, respectively.

In left ventriculography, mitral regurgitation severity is assessed visually by the amount of contrast ejected into the left atrium during systole. In echocardiography, the velocity across the valve is assessed to determine the severity of mitral regurgitation. In addition, color Doppler imaging is similar to MRI in that both the modalities are looking at turbulent flow. However, compared to MRI, echocardiography looks at the velocity of both laminar and turbulent flow in assessing the severity of mitral regurgitation.

Flow velocity mapping and volume analysis are two other MRI techniques used in assessing the severity of mitral regurgitation. Neuhold et al., evaluated 46 patients with angiographically confirmed regurgitant lesions and found a good correlation with MRI using the volume analysis method. [20] In another study, Fujita et al., compared color Doppler echocardiography to velocity encoded cine MRI in grading mitral regurgitation and found that regurgitant fraction obtained by MRI correlated well with the echocardiographic severity of mitral regurgitation, r = 0.87. [21] Although both flow velocity mapping and volume analysis have been validated in previous studies, there are certain limitations to these techniques. They both require additional pulse sequences and flow velocity mapping requires fairly extensive time-consuming post processing. Evaluation of mitral regurgitation by visual assessment using a routine pulse sequence (SSFP) offers a simple alternative, particularly as SSFP is used on a routine basis in the standard evaluation of the heart. Therefore, developing a simple grading system, i.e. specific visual parameters for routine clinical use, will provide an easier and less time consuming tool for assessing mitral regurgitation severity.

### Limitations

Mitral regurgitation is dependent upon the volume status and blood pressure of patients, which may potentially change in between each imaging study. We were unable to completely account for all loading conditions; however, the close proximity of these imaging tests minimizes this potential limitation.

This study is an attempt to validate the proposed visual assessment criteria for the severity of mitral regurgitation and has not been studied previously. We did not use a reference method to look at the reliability of cine MRI. Instead, comparison was made with the standard imaging methods. Larger clinical trials may be required to further validate the criterion before being used in the clinical setting.

SSFP sequence utilized to assess severity of mitral regurgitation has an inherently short echo time (TE). Using short TE sequences may not allow for adequate spin dephasing and potentially may decrease the accuracy of the test.

Severity of mitral regurgitation was obtained from consensus of three cardiologists, interpreting the images at same time, and we could not obtain the inter-observer variability. Similar method was followed for interpreting cardiac catheterization and Doppler echocardiography images.

Another possible limitation may be selection bias in finding patients who had all 3 tests, which included patients with heart failure and those undergoing viability and coronary artery disease assessment, as compared to the general valve disease population. Theses findings may be less applicable to patients not selected for multiple imaging procedures.

### Conclusion

In order to determine the need and success of medical therapy, as well as, to decide on the correct timing for surgery, prudent assessment of valvular regurgitation is imperative. Cine MRI offers a reasonable alternative to both Doppler echocardiography and to a fair extent cardiac catheterization for assessing the severity of mitral regurgitation with specific visual parameters during routine clinical cardiac MRI.
